# Identification of gene targets that potentiate the action of rifampicin on Mycobacterium bovis BCG

**DOI:** 10.1099/mic.0.001488

**Published:** 2024-08-16

**Authors:** Pooja Chand, Tom A. Mendum, Rachel E. Butler, Suzanne M. Hingley-Wilson, Graham R. Stewart

**Affiliations:** 1 Department of Microbial Sciences, School of Biosciences, Faculty of Health and Medical Sciences, University of Surrey, Guildford, GU2 7XH United Kingdom

**Keywords:** *Mycobacterium tuberculosis*, ParB, PknE, rifampicin, Rnase HI, TnSeq, transposon sequencing

## Abstract

Tuberculosis (TB) caused by bacteria of the *Mycobacterium tuberculosis* complex remains one of the most important infectious diseases of mankind. Rifampicin is a first line drug used in multi-drug treatment of TB, however, the necessary duration of treatment with these drugs is long and development of resistance is an increasing impediment to treatment programmes. As a result, there is a requirement for research and development of new TB drugs, which can form the basis of new drug combinations, either due to their own anti-mycobacterial activity or by augmenting the activity of existing drugs such as rifampicin. This study describes a TnSeq analysis to identify mutants with enhanced sensitivity to sub-minimum inhibitory concentrations (MIC) of rifampicin. The rifampicin-sensitive mutants were disrupted in genes of a variety of functions and the majority fitted into three thematic groups: firstly, genes that were involved in DNA/RNA metabolism, secondly, genes involved in sensing and regulating mycobacterial cellular systems, and thirdly, genes involved in the synthesis and maintenance of the cell wall. Selection at two concentrations of rifampicin (1/250 and 1/62 MIC) demonstrated a dose response for mutants with statistically significant sensitivity to rifampicin. The dataset reveals mechanisms of how mycobacteria are innately tolerant to and initiate an adaptive response to rifampicin; providing putative targets for the development of adjunctive therapies that potentiate the action of rifampicin.

## Introduction

Rifampicin is one of the frontline antibiotics used to treat tuberculosis (TB). It is responsible for exerting bactericidal activity through the inhibition of the early stages of gene transcription by binding to the β-subunit of RNA polymerase (RpoB) which is encoded by the *rpoB* gene in mycobacteria [1]. It has considerable hepatotoxicity but is usually administered as part of a multidrug therapy for 6 months for effective treatment of active TB, and on occasion as a single antibiotic for 4 to 6 months to treat latent tuberculosis. The WHO includes rifampicin on its list of essential medicines [2]. However, there are worrying rates of rifampicin resistance in *Mycobacterium tuberculosis* strains worldwide. In the year 2021, there were approximately 450 000 new cases of multidrug-resistant (MDR) TB globally, which by definition involves rifampicin and isoniazid resistance. The WHO have set goals to eliminate TB infection by 2035, however this issue cannot be fulfilled without addressing antibiotic resistance [3]. An adjunctive therapy which would work alongside rifampicin to shorten its duration of treatment or lower its minimum inhibitory concentration (MIC) would be of significant benefit because this may lead to improved treatment outcomes and a lower frequency of mutation conferring *de novo* rifampicin resistance.

One approach to identify new drugs that work in combination is to identify potentiation or synergism between existing drug compounds, and medium/high-throughput *in vitro* and *in vivo* screens have been developed to achieve this [4,7]. A rational target-based approach to develop new drugs that potentiate the action of rifampicin would be greatly facilitated by identifying mycobacterial genes which have an interaction with rifampicin activity. However, the experimental validation and identification of individual gene-drug interactions is time-consuming because of the slow growth and protracted pace of genetic manipulation of mycobacteria. The TnSeq procedure involving high-density transposon mutagenesis and tracking of mutant pools by parallel sequencing partially overcomes these issues, providing a powerful tool to screen whole genomes for fitness phenotypes in different selective environments [8,10]. Two excellent studies have used TnSeq in mycobacteria to examine genetic interactions with antibiotic exposure [11,12]. The study by Xu grew *M. tuberculosis* in broth medium and identified mutations that rendered the bacterium more sensitive to a range of antibiotics [11]. Interestingly, there was considerable overlap between the gene interactions with rifampicin and several other antibiotic classes. The study identified the cell wall to be a major determinant of rifampicin susceptibility but surprisingly did not identify any efflux pumps. The study by Bellerose selected rifampicin sensitive mutants in murine infection and also found a preponderance of cell wall mutants implicating permeability as the dominant intrinsic resistance factor in this environment [12]. However, there was <40% overlap between the individual gene-rifampicin interactions identified in the two studies, suggesting that different selection environments can highlight different aspects of what makes a mycobacterium innately resistant to rifampicin. Further information on genes important to rifampicin sensitivity is provided from a genome-wide CRISPRi study of *M. tuberculosis* exposed to various antibiotics including rifampicin in broth media [13]. The advantage of this latter approach is the capability to examine interactions with essential genes.

To provide an alternative set of conditions that may reveal other genes important to rifampicin susceptibility, the present study selects a *Mycobacterium bovis* Bacille Calmette-Guérin (BCG) transposon mutant library on solid media containing concentrations of rifampicin below the MIC, calibrated to kill ~40 and 90 % of the bacteria. These antibiotic selective pressures in an environment without cell wall stress from shear forces, detergent or immunity, revealed novel gene interactions with rifampicin which identify putative drug targets for adjunctive and synergistic therapies to make rifampicin more effective.

## Methods

### Bacteria and growth conditions

*Mycobacterium bovis* BCG Pasteur (University of Surrey culture collection) was routinely cultured at 37 °C on Middlebrook 7H11 plates containing 10% oleic acid/albumin/dextrose/catalase (OADC) enrichment and 0.5% glycerol or in Middlebrook 7H9 broth containing 10% ADC, 0.2% glycerol and 0.05% Tween80. Kanamycin at 20 µg µl^−1^ was added to media for selection of transposon mutants.

### Transposon mutagenesis of *M. bovis* BCG with φMycoMarT7

The *M. bovis* BCG MycoMar transposon mutant library was constructed as previously described [14]. Briefly, 100 ml of late log *M. bovis* BCG shaken culture was washed twice in MP buffer (50 mM Tris-HCl, pH 7.5, 150 mM NaCl, 10 mM MgSO4, 2 mM CaCl2) at 37 °C by centrifugation at 4000 ***g*** for 12 min, and then incubated with a minimum of 1×10^11^ pfu of ɸMycoMarT7 phage for 4 h. The φMycoMarT7 stock was generated as previously described by amplification in *M. smegmatis* [15]. The suspension was washed and recovered by centrifugation and plated on ten 15 cm 7H11 plates with kanamycin. The number of mutants generated in the transduction was enumerated by serial dilution and plating on 7H11 with kanamycin for colony forming unit (c.f.u.) determination. After 3–4 weeks the library was harvested from the plates and aliquots frozen at − 80 °C in 15% glycerol in water.

### Determination of sub-MIC levels of rifampicin in BCG Pasteur

Rifampicin stock solutions were made by diluting rifampicin in dimethyl sulfoxide (DMSO) (Sigma-Aldrich) to reach a concentration of 100 µg ml^−1^. The stock solutions were stored in the −20 °C freezer. Serial four-fold dilutions of the rifampicin stock solution were added to aliquots of 7H11 to give final antibiotic concentrations of 8000 ng ml^−1^ to 2 ng ml^−1^ (seven dilutions in total). These are the critical concentrations of rifampicin recommended by the Clinical and Laboratory Standards Institute (CLSI) for *M. tuberculosis* susceptibility testing. Ten-fold serial dilutions down to 10^−6^ of mid log phase *M. bovis* BCG broth culture were plated on the 7H11 media with the various rifampicin concentrations. The experiment was performed in triplicate with a growth control (no rifampicin). The cultures were incubated at 37 °C for 2–3 weeks before the colonies were counted to determine the level of killing exhibited by the various rifampicin concentrations. Rifampicin concentrations giving approximately 5 and 90% growth inhibition of BCG were chosen for the TnSeq screen.

### Selection of BCG Tn mutant library in the presence of sub-MIC rifampicin concentrations

A 500 µl aliquot of the BCG transposon mutant library containing approximately 5×10^7^ c.f.u. was spread onto 15 cm 7H11 agar plates with the either the sub-MIC rifampicin concentrations (8 and 2 ng ml^−1^) or no rifampicin as a control and grown for 3 weeks at 37 °C. Four independent selections were made for each treatment. The mutants that survived were harvested by scraping from agar plates with the addition of 10 ml Tris-EDTA buffer and centrifuged at 4000 r.p.m. for 20 min and the supernatant was discarded. The pellet was stored in the −80 °C freezer prior to the isolation and extraction of genomic DNA. Four replicate selections were performed.

### Purification of gDNA from transposon libraries for TnSeq

The BCG pellet was resuspended in 4.5 ml Glucose-Tris-EDTA (GTE) and 10 mg ml^−1^ lysozyme (Sigma Aldrich) and incubated for 2 h at 37 °C. One millilitre of 10% sodium dodecyl sulphate (SDS) and 500 µl of 10 mg ml^−1^ proteinase K (Sigma Aldrich) were added and the solution was incubated at 55 °C for 40 min, followed by the addition of 2 ml of 5M NaCl and 1.6 ml of cetrimide saline solution (CTAB) and a further incubation period of 10 min at 65 °C.

The lysate was then extracted with chloroform-isoamyl alcohol (2–3 times), followed by precipitation of nucleic acids with one-tenth volume of 3M sodium acetate and 1 vol of isopropanol. The DNA pellet was washed with 70% ethanol, air dried and then reconstituted in 50 µl of TE buffer. The extracted gDNA was run on a 0.7% agarose gel to visualize the presence of gDNA. Each 50 µl of gDNA sample was treated with 1 µl of RNAse, (DNAse free, Sigma Aldrich), cleaned up using a Qiaquick PCR Purification kit (Qiagen) and the purified gDNA was quantified using a Nanodrop 2000 UV-Vis spectrophotometer.

### Preparation of TnSeq-Illumina sequencing libraries

A 5 µg aliquot of the gDNA from each of the eight mutant pools was added to nuclease free water to achieve a total volume of 87 µl in a microTUBE AFA Fibre Pre-Slit Snap-Cap (Covaris). The Covaris Focused-ultrasonicator S220 machine was used to shear DNA to approximately 500 bp using the following settings: duty factor – 5%, peak incident power – 105, 200 cycles per burst for 80 s at a temperature of 7 °C. To ensure that the DNA fragments had been sheared to 500 bp, the DNAs were run on a 1% agarose gel.

The sheared ends of the DNA were repaired, 5′ phosphorylated and 3′ dA-tailed using the NEBNext Ultra II End Repair/dA-Tailing Module (New England Biolabs). Then 26 µl of sheared DNA was mixed with 3 µl of NEBNext Ultra II End Prep Enzyme Mix and 7 µl of NEBNext Ultra II End Prep Reaction Buffer and 34 µl of nuclease free water to achieve a final volume of 70 µl. The reaction mixture was then incubated in a thermocycler for 30 min at 20 °C followed by a 30 min incubation at 60 °C. The reaction mixture was cleaned up using 1.0 X volume of Agencourt AMPure XP beads and eluted in 50 µl of nuclease free water. Following this, adapters were prepared which have a 3′ T overhang to ligate to the A-tailed DNA fragments.

The two adapters used were; Adap one and Adap two (Table S1, available in the online version of this article). Adap one and Adap two were prepared by adding equimolar volumes (100 µM) of each adapter in 2 mM MgCl_2_, which gave a final concentration of 50 µM of each adapter. The mixture was heated at 95°C for 5 min followed by slow cooling at room temperature for 2–3 h. The annealed adapters were then ligated to the A-tailed ends of gDNA using Blunt/TA Ligase Master mix (NEB, UK). This was done by mixing ten-fold molar ratios of the adapter mix to the dA-tailed library and adding an equal volume of Blunt/TA Ligase Master mix and incubating the reaction mixture for 1 h at RT. After the incubation step, each reaction was cleaned up using 1.0 X volume of Agencourt AMPure XP beads and eluting the samples in 50 µl of nuclease free water.

Transposon junctions were amplified using the primers; IS6 and Mar A to Mar L (Table S1). The primers contained a P5-index that identifies the sample and a random P7-index to allow PCR-generated artefacts to be identified and removed from the data. The reaction mixture consisted of 25 µl master mix, 2.5 µl IS6 primer, 2.5 µl of Himar-1 MycoMarT7 mariner transposon primer, 5 µl template, 15 µl nuclease free water. Control reactions of 10 µl with IS6 primer/template only and IS6/MarX primer mix with no template were used. Following the amplification step, samples were pooled and purified using 1.0 X volume of Ampure XP beads and eluted in 50 µl water. The fragment size was assessed using gel electrophoresis on a 0.8% agarose gel and Agilent 2100 Bioanalyser to check for a desired fragment length of ~500 bp. DNA concentration was quantified using a Quantus Fluorometer (Promega). The purified and indexed samples were mixed and combined in equimolar concentrations to give the desired volume of 15 µl with a DNA concentration of 50 ng µl^−1^. Transposon junctions were sequenced on an Illumina HiSeq 2500 platform using a single index and a single read of 100 cycles for Illumina sequencing (Genewiz). Sequencing files were deposited at Bioproject PRJNA1112803.

### Data analysis

In the initial step, data was demultiplexed using the P5 index and quality controlled and aligned essentially as previously described [15,16]. Reads were mapped onto the reference genome (*M. bovis* BCG Pasteur 1173) using Bowtie and only kept if they had a mapping quality of >30. It was assumed that any reads with identical P7 index reads mapping to the same TA site and DNA sequences were duplicates of PCR with random code sequences and were therefore counted only once.

To identify the relative abundance/rifampicin-sensitivity of mutants in untreated and rifampicin-treated pools of mutants we used the TRANSIT-2 resampling method [17]. The read counts were normalised using Trimmed Total Reads (TTR) normalization and *p* values adjusted for multiple comparisons (q-value) using the Benjamin-Hochberg correction.

## Results

### Selection of a BCG Pasteur transposon mutant library on sub-MIC of rifampicin

**Fig. 1.**
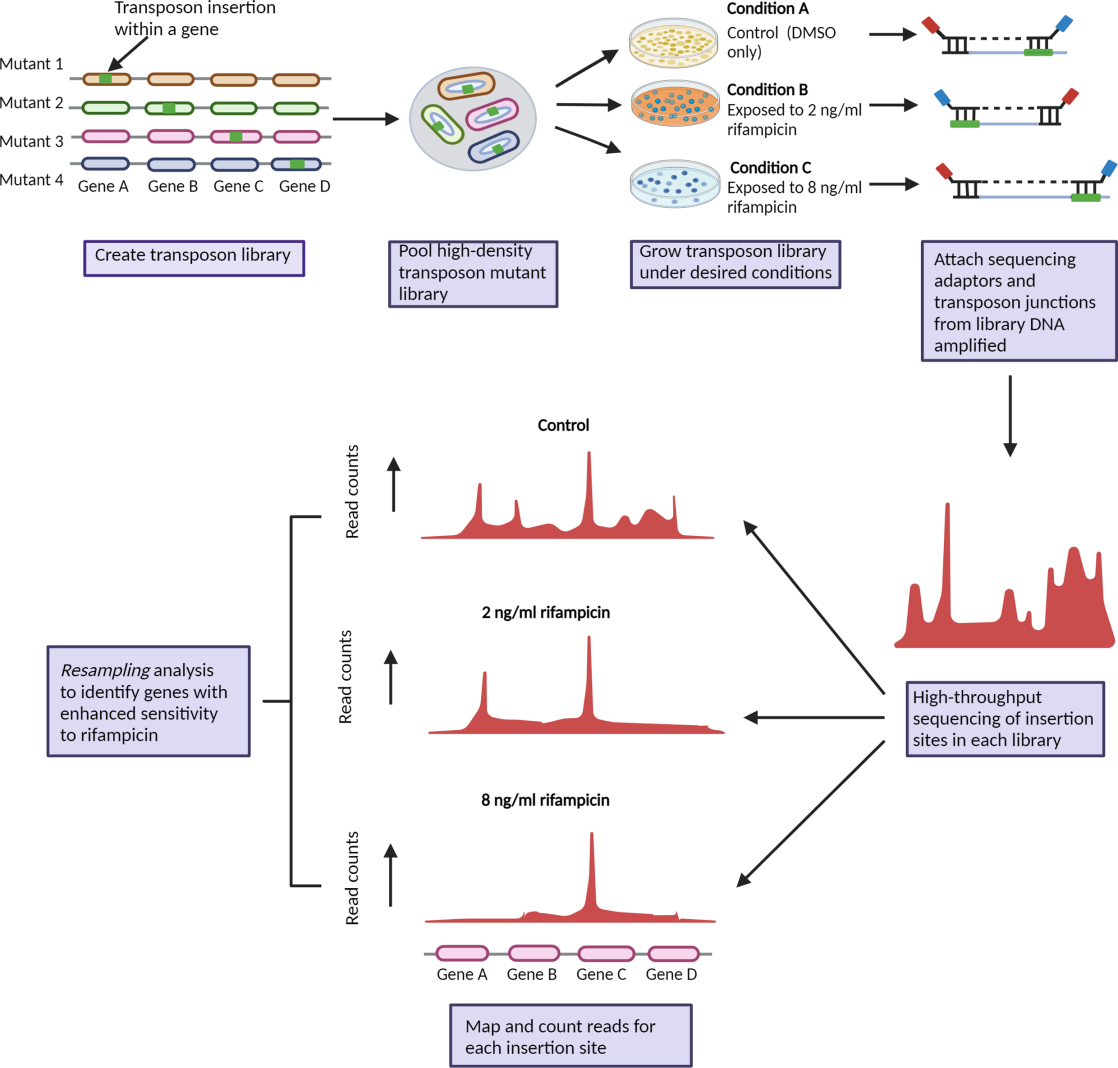
Experimental strategy to identify *M. bovis* BCG genes that interact with rifampicin treatment and are required for intrinsic resistance. A MycoMar transposon library was generated in *M. bovis* BCG and selected on 7H11 solid media containing sub-MIC concentrations of rifampicin. The identity and abundance of mutants is revealed by TnSeq and compared between rifampicin treated and non-treated pools of mutants.

**Fig. 2.**
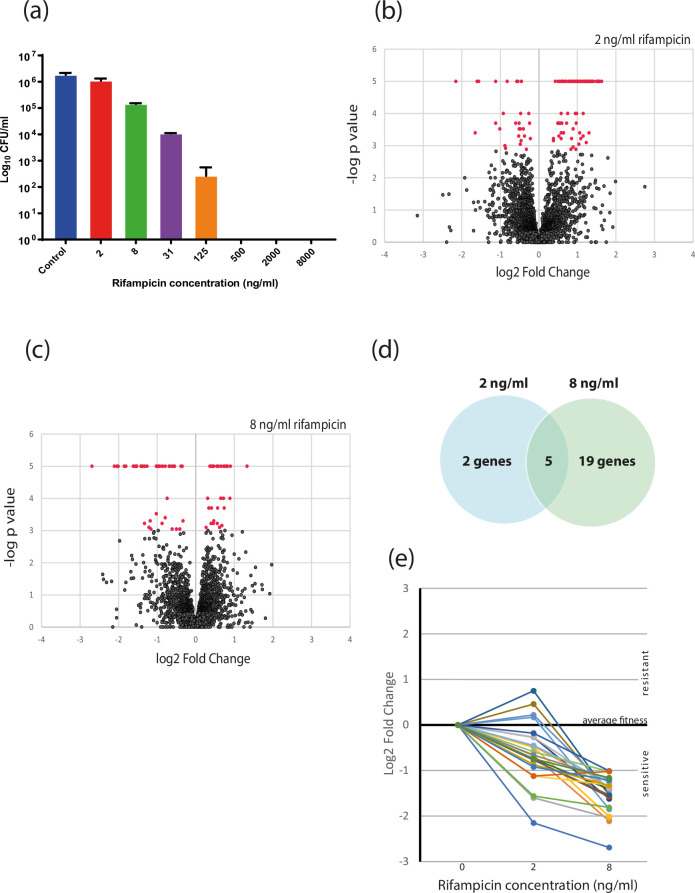
Selection of rifampicin-sensitive *M. bovis* BCG mutants. (**a**) Determination of minimum inhibitory concentration (MIC) and sub-MIC of rifampicin for *M. bovis* BCG. Bacterial cultures were serially diluted, and spot dilutions were made on 7H11 agar with the respective rifampicin concentrations. Colonies were enumerated after 3 weeks. Graph shows the mean of three biological replicates and error bars are one SD. (**b**) and (**c**), TnSeq assessment of BCG mutant fitness in sub-MIC rifampicin. Volcano plots show the relationship between fold change and fdr-adjusted *P* values derived by the TRANSIT Resampling test of TnSeq reads from pools of BCG mutants selected on 2 ng ml^−1^ and 8 ng ml^−1^ rifampicin respectively. Mutations in genes with an adjusted *P* value<0.05 were deemed to have significantly different survival/fitness are coloured red. (**d**) Overlap in mutants with significantly increased sensitivity to rifampicin at 2 and 8 ng ml^−1^. (**e**) Dose response of rifampicin sensitive mutants. The fold change in survival at 2 ng ml^−1^ and 8 ng ml^−1^ rifampicin was plotted for all mutants (n=24) which had at least a two-fold reduction on 8 ng ml^−1^ rifampicin and an adjusted *P* value<0.05.

The primary aim of this study was to select a mycobacterial transposon mutant library in the presence of rifampicin to identify transposon mutants that had enhanced susceptibility to rifampicin. An overview of the experimental strategy is shown in Fig. 1. To achieve this, we first determined sub-MIC concentrations of rifampicin which provided a sufficient selective pressure on the library but without killing all the bacteria. Survival/growth of BCG on 7H11 solid medium containing various concentrations of rifampicin is shown in Fig. 2a. The MIC in these conditions was determined to be 500 ng ml^−1^. The viable count of BCG was reduced by 40, 92 and 99% in 2 ng ml^−1^, 8 ng ml^−1^ and 31 ng ml^−1^ rifampicin respectively. We chose to select the BCG mutant library on 7H11 containing 2 ng ml^−1^ and 8 ng ml^−1^ rifampicin, with the view that these would provide the dynamic range to capture even subtle changes in susceptibility.

Aliquots of BCG transposon mutant library stock were cultured for 3 weeks on 7H11 agar plates with rifampicin concentrations 2 ng ml^−1^ and 8 ng ml^−1^ and without rifampicin. Colonies were harvested and genomic DNA was extracted from each pool for sequencing of transposon insertion sites. Sequencing files are available at Bioproject PRJNA1112803. Each of the libraries was shown to have approximately 30 000 unique transposon inserts/mutants, as assessed post-processing (Table S2). The *M. bovis* BCG genome contains ~73 902 TA dinucleotide sites. The calculated number of TA sites hit by the mycomar transposon and reported by sequencing was between approximately 38.7 and 43.2% of the total number of TA sites for all sequenced transposon mutant pools (Table S2). Moreover, between 77.6 and 79.7% of all BCG genes were found to contain at least one transposon insertion, which is close to the previously determined number of all non-essential genes. On average, slightly fewer mutated genes were detected in the rifampicin treated pools, but this only reached statistical significance for the 2 ng ml^−1^ rifampicin selections. This BCG mutant library was previously analysed to estimate gene essentiality and determined 643 essential genes [14]. Here, consistent with this finding, we identified 674 genes in the unselected control group without any transposon insertion.

### Mutants with altered sensitivity to sub-MIC rifampicin

Comparison of the mutant pools selected in rifampicin with pools grown for an equal length of time without rifampicin was achieved by TRANSIT Resampling analysis of sequenced transposon insertion sites (Tables S3 and S4). In the lowest concentration of rifampicin, 2 ng ml^−1^, 42 mutants were found to have significantly different abundance compared to the control of which only seven were significantly less abundant indicative of increased sensitivity to rifampicin (Fig. 2b and Table 1). In 8 ng ml^−1^ rifampicin, 25 mutants were identified to have an altered abundance, 24 of which show a decreased survival in rifampicin (Fig. 2c and Table 2). Five of the seven rifampicin sensitive mutants identified at 2 ng ml^−1^ were also identified at 8 ng ml^−1^ rifampicin with four mutants showing an exacerbated decrease in survival in the higher dose antibiotic selection (Fig. 2d). We further examined the rifampicin dose effect of the selections by plotting the fold change in survival at 2 ng ml^−1^ and 8 ng ml^−1^ rifampicin for all mutants (*n*=24) which had at least a two-fold reduction on 8 ng ml^−1^ rifampicin and an adjusted *P* value<0.05. The majority of these mutants showed a clear dose effect with sensitivity to 2 ng ml^−1^ rifampicin which becomes more pronounced at 8 ng ml^−1^ rifampicin (Fig. 2e). This provided a strong indication that it was rifampicin pressure driving the fitness decrease observed in these mutants.

**Table 1.** *Mycobacterium bovis* BCG mutants with significantly altered survival upon exposure to rifampicin at 2 ng ml^−1^ on Middlebrook 7H11 agar (Log2FC>1 or <-1, adjusted *P* value<0.05)

Gene locus	Gene name	*Mtb* H37Rv ortholog	Description	Log 2 FC	Adj *P* value
BCG_1782	*pknE*	Rv1743	Putative transmembrane serine/threonine-protein kinase E. Signal transduction	−2.15	<0.001
BCG_0012	–	Rv0012	Hypothetical protein	−1.65	0.018
BCG_1456	–	Rv1395	Transcriptional regulatory protein for Cyp132	−1.6	<0.001
BCG_3797	*thrE*	Rv3737	Putative transporter	−1.56	<0.001
BCG_1246 c	*chp2*	Rv1184c	Chp2 polyacyltrehalose synthesis	−1.12	<0.001
BCG_3331 c	*glpD2_1*	Rv3302c	Putative glycerol-3-phosphate dehydrogenase	−1.12	0.010
BCG_2672 c	–	Rv2660c	Hypothetical protein	−1.01	0.014
BCG_0258	–		Hypothetical protein	1.04	<0.001
BCG_2967 c	*lppX*	Rv2945c	Putative lipoprotein	1.04	0.035
BCG_0510 c	*pcaA*	Rv0470c	Mycolic acid synthase	1.06	<0.001
BCG_2941 c	*amt*	Rv2920c	Putative ammonium-transport integral membrane protein amt	1.06	0.018
BCG_2966	–	Rv2943A	Putative transposase	1.09	<0.001
BCG_3597 c	*PPE62*	Rv3533c	PPE family protein	1.09	0.025
BCG_0924 c	*PE_PGRS15*	Rv0872c	PE-PGRS family protein	1.14	<0.001
BCG_2996 c	–	Rv2974c	Hypothetical protein	1.15	<0.001
BCG_0356	–	Rv0316	Putative muconolactone isomerase	1.15	0.006
BCG_0218 c	–	Rv0181c	Hypothetical protein	1.15	0.022
BCG_2995 c	*recG*	Rv2973c	Putative atp-dependent DNA helicase, DNA repair	1.17	<0.001
BCG_3211	–	Rv3189	Hypothetical protein	1.17	<0.001
BCG_2977	–	Rv2956	Hypothetical protein	1.19	<0.001
BCG_2991 c	*lipN*	Rv2970c	Putative lipase/esterase	1.2	<0.001
BCG_2988 c	*pca*	Rv2967c	Pyruvate carboxylase, gluconeogenesis	1.21	<0.001
BCG_2971 c	*fadD29*	Rv2950c	Acyl-CoA synthetase, PGL synthesis	1.22	<0.001
BCG_2972 c	–	Rv2951c	Putative oxidoreductase	1.22	<0.001
BCG_2633	*PPE42*	Rv2608	PPE family protein	1.23	0.032
BCG_2984	–	Rv2963	Putative integral membrane protein	1.24	<0.001
BCG_2982	–	Rv2961	Putative transposase	1.25	<0.001
BCG_2975 c	–	Rv2954c	Hypothetical protein	1.26	<0.001
BCG_2983 c	–	Rv2962c	Putative glycosyl transferase	1.3	<0.001
BCG_2993	–	Rv2971	Putative oxidoreductase	1.3	0.018
BCG_2990 c	–	Rv2969c	Putative membrane or secreted protein	1.31	<0.001
BCG_2969 c	*fadD22*	Rv2948c	Acyl-CoA synthetase, PGL synthesis	1.32	<0.001
BCG_2968 c	*pks1*	Rv2946c	Putative polyketide synthase	1.34	<0.001
BCG_2994 c	–	Rv2972c	Putative membrane or exported protein	1.36	<0.001
BCG_2974	–	Rv2953	Hypothetical protein	1.38	<0.001
BCG_2979 c	–	Rv2958c	PGL/p-HBAD biosynthesis glycosyltransferase	1.38	<0.001
BCG_2989 c	–	Rv2968c	Putative integral membrane protein	1.42	<0.001
BCG_2973	–	Rv2952	Putative methyltransferase	1.5	<0.001
BCG_3899	–	Rv3836	Hypothetical protein	1.5	<0.001
BCG_2985	*purU*	Rv2964	Formyltetrahydrofolate deformylase, purine synthesis	1.54	<0.001
BCG_2976 c	–	Rv2955c	Hypothetical protein	1.57	<0.001
BCG_2980 c	–	Rv2959c	Putative methyltransferase	1.63	<0.001

**Table 2.** *Mycobacterium bovis* BCG mutants with significantly altered survival upon exposure to rifampicin at 8 ng ml^−1^ on Middlebrook 7H11 agar (Log2FC>1 or <-1, adjusted *P* value<0.05)

Gene locus	Gene name	*Mtb* H37Rv ortholog	Description	Log 2 FC	Adj P value
BCG_1782	*pknE*	Rv1743	Putative transmembrane serine/threonine-protein kinase E. Signal transduction	−2.69	<0.001
BCG_3073 c	–	Rv3049c	Putative monooxygenase	−2.11	<0.001
BCG_1456	–	Rv1395	Transcriptional regulatory protein for Cyp132	−2.04	<0.001
BCG_3328 c	*atsB*	Rv3299c	Putative arylsulfatase	−2.01	<0.001
BCG_0021	*cwlM*	Rv3915	Peptidoglycan synthesis regulator	−1.85	<0.001
BCG_3797	*thrE*	Rv3737	Putative transporter	−1.81	<0.001
BCG_1041	–	Rv0986	Putative adhesion component atp-binding protein ABC transporter	−1.62	<0.001
BCG_0380	*iniB*	Rv0341	Isoniazid inductible gene protein	−1.58	<0.001
BCG_0023 c	*parB2*	Rv3917c	Putative chromosome partitioning protein	−1.54	<0.001
BCG_1274 c	*PE14*	Rv1214c	PE family protein	−1.53	<0.001
BCG_2260	*fabD*	Rv2243	Malonyl CoA-acyl carrier protein transacylase	−1.53	<0.001
BCG_2151 c	–	Rv2134c	Hypothetical protein	−1.42	<0.001
BCG_2246 c	*rnhC*	Rv2228c	Bifunctional RNase H/acid phosphatase	−1.41	<0.001
BCG_1676	*uvrA*	Rv1638	Excinuclease ABC subunit A, DNA repair	−1.39	<0.001
BCG_0201 c	*mce1R*	Rv0165c	Mce1R transcriptional regulator, transport	−1.37	<0.001
BCG_3684 c	–	Rv3626c	Hypothetical protein	−1.33	0.036
BCG_1246 c	*chp2*	Rv1184c	Chp2 polyacyltrehalose synthesis	−1.32	<0.001
BCG_2781 c	*thyA*	Rv2764c	Thymidylate synthase	−1.26	<0.001
BCG_0018	*rsmA*	Rv3912	RsmA anti- SigM, transcription regulator	−1.21	0.046
BCG_0200	*TB18.5*	Rv0164	Predicted outer membrance protein	−1.18	0.033
BCG_1581	*fadD24*	Rv1529	Acyl-CoA synthetase	−1.16	0.049
BCG_0485 c	–	Rv0446c	Putative transmembrane protein	−1.02	<0.001
BCG_3331 c	*glpD2_1*	Rv3302c	Putative glycerol-3-phosphate dehydrogenase	−1.02	0.021
BCG_1863	–	Rv1828	Transcriptional regulator	−1.01	<0.001
BCG_0509	*umaA1*	Rv0469	Putative mycolic acid synthase	1.33	<0.001

#### Cell wall mutants sensitive to rifampicin

Genes in which mutation induces sensitivity to rifampicin might include those encoding cellular architecture or defence systems which are omni-present in the bacterium and do not require induction. The most obvious example would be genes involved in synthesis and maintenance of the cell wall, the major physicochemical barrier to drug permeability. The present study identified *chp2* (BCG_1246 c; Rv1184c), which encodes an acyltransferase, essential for synthesis of outer membrane polyacyltrehalose (PAT), as required for intrinsic resistance to rifampicin at both drug concentrations. This gene was previously shown to be important for rifampicin tolerance during murine infection [12]. Further evidence implicating the importance of the cell wall was provided by the increased sensitivity of mutants in *fabD* (BCG-2260; Rv2243), which encodes an essential role in mycolic acid biosynthesis. Although this gene is essential, our analysis included insertional mutants towards the 3′ end of the gene, which resulted in viable mutants which must have some detrimental effect on gene function and act synergistically with rifampicin. In support of the vital role of *fabD* in rifampicin resistance, this was one of only four proteins found to be overtly reduced following rifampicin treatment of *M. tuberculosis* [18]. Indeed, the importance of cell wall remodelling during rifampicin exposure is also indicated by the finding in the present study that the transcriptional repressor of the Mce1 lipid transport system, Mce1R (BCG_0201 c; Rv0165c), is required for innate tolerance to rifampicin. Other studies have demonstrated that mutants in the Mce1 system components have increased drug tolerance in phosphate starved and stationary phase *M. tuberculosis* [19]. Interestingly, mutation of the duplication region DU1-positioned CwlM peptidoglycan synthesis regulator gene [20] decreased bacterial fitness in rifampicin, further implicating changes in cell wall permeability as a central factor in rifampicin susceptibility. This gene is essential in most strains of *M. tuberculosis/M. bovis* but BCG Pasteur is a natural merodiploid for genes in the DU1 duplication region which spans the chromosomal origin of replication and so allows TnSeq to report on mutation on one copy of the gene. Also demonstrated to be necessary for rifampicin tolerance was the *iniB* gene (BCG_0380; Rv0341) (Isoniazid inductible gene protein IniB), which together with the coexpressd *iniA* and *iniC* appears to be involved in membrane stabilization [21].

#### Transporter mutants associated with rifampicin sensitivity

It is interesting to note the absence of obvious drug efflux systems [22] in the identified rifampicin susceptible mutants. However, mutants in two transport systems were more susceptible to rifampicin. BCG_1041/Rv0986 encodes an ATP-binding cassette (ABC transporter) involved in an export function [23] and transposon insertion in this gene was identified as conferring increased sensitivity to rifampicin. It is thought to be involved in active transport of a molecule involved in adhesion to host cells across the mycobacterial cell wall and may also be involved in attachment and virulence itself [24]. Rv0986 comprises one part of a three gene operon from Rv0986 to Rv0988 and is present in pathogenic mycobacteria only [25,26]. As an inner membrane exporter, it could be directly involved in export of antibiotics or cell wall components that are important for resistance to rifampicin.

A further transporter, the threonine transporter, ThrE (BCG-3797; Rv3737), also appears to be associated with rifampicin tolerance and mutants had significantly increased sensitivity at both 2 ng ml^−1^ and 8 ng ml^−1^ rifampicin. The repertoire of molecules transported by ThrE has not been revealed for mycobacteria, but *thrE* deletion mutants have an altered cell morphology [27] suggesting that drug sensitivity may be associated with changes in cell wall structure rather than drug efflux.

#### Adaptation and stress response genes needed for rifampicin tolerance

Aside the omni-present innate resistance factors, drug tolerance can be achieved via adaptation to the deleterious effects of the antibiotic. For example, intracellular signalling cascades catalysed by the serine threonine protein kinase PknG have a significant role in antibiotic sensitivity to multiple drugs including rifampicin via PknG activity to regulate central carbon and nitrogen metabolism by phosphorylation of its substrates or by phosphorylation of the metabolic regulator GarA [11,28, 29]. PknG was not detected in the present study but mutants in the PknE kinase (BCG_1782; Rv1743) were found to be more susceptible to rifampicin at both 2 ng ml^−1^ and 8 ng ml^−1^. PknE is a receptor kinase with the capacity to sense the extracellular environment and, like PknG, PknE is important for manipulation of host macrophage biology and intracellular survival of *M. tuberculosis* [30,31]. It is not known which stimuli PknE has the capacity to sense, nor the repertoire of phosphorylation substrates to which it transduces the signal. The prospect that PknE has a role in sensing low concentrations of rifampicin or its activity and effecting adaptive drug tolerance is consistent with the observation that PknE is upregulated over five-fold after exposure of the bacterium to rifampicin [32].

Several other regulatory systems may provide tolerance-inducing adaptation to rifampicin (BCG-1455/Rv1395: BCG_1863/Rv1828: *rsmA* BCG_0018/Rv3912: MceR1). Mutants in the transcriptional regulator BCG-1455/Rv1395 were indicated to be sensitive to both rifampicin concentrations and this regulator is essential for control of the Cyp132 cytochrome P450 [33]. RsmA is the anti-sigma-factor for σM, which controls the cell wall transport system ESX4.

RNase HI is an enzyme which removes the DNA/RNA hybrids known as R-loops, which are formed during transcription and its activity is essential for optimal growth. Previous studies have demonstrated that RnhC (BCG_2246 c; Rv2228c) is the sole RNase H1 in *M. tuberculosis* and it has been assumed that mutation of the gene is completely non-viable precluding study of null mutants. Here however, transposon mutants of RnhC were detectable in the mutant library and their fitness was quantifiable by TnSeq allowing determination that RnhC is essential to provide intrinsic resistance against rifampicin. Indeed, this provides direct evidence in an *M. tuberculosis* complex species to support previous studies in *M. smegmatis*, which has two RNase H1 enzymes, showing that sub-MIC concentrations of rifampicin induce the accumulation of R-loops and that depletion of RNase H1 significantly potentiates the action of rifampicin [34].

## Discussion

The importance of combining antibiotics to produce synergistic effects on efficacy has been recognized since the 1950s. To identify molecules, mechanisms and subcellular structures that might be targeted to potentiate the action of rifampicin, we investigated drug-gene interactions in *M. bovis* BCG by TnSeq assessment of mutant sensitivity to sub-inhibitory levels of rifampicin. The mode of action of rifampicin is to inhibit transcription by binding to the β subunit of RNA polymerase [1] so blocking the elongation step therefore preventing the ultimate synthesis of proteins which further leads to the killing of mycobacteria [35,36]. There is ample evidence from the literature that mutations in the *M. tuberculosis* β subunit of the RNA polymerase gene *rpoB* are the most frequent mechanism of drug-resistance against rifampicin [37]. However other mechanisms have been implicated in rifampicin resistance, such as, enhanced activity of efflux pumps, metabolic shifts and regulatory changes of the *rpoB* gene [38,40]. Despite the variety of mechanisms potentially involved in ‘genetic’ rifampicin resistance, whole genome mutant screens performed in liquid broth and murine infection identified innate resistance mechanisms were dominated by molecules involved in synthesis and maintenance of cell wall structures [11,12]. Thus, impermeability appeared to be an important factor for innate resistance to rifampicin. There was, however, only a limited overlap between these studies suggesting that tolerance to rifampicin was in part dependent on the environment. To dig deeper into the tolerance mechanisms beyond cell wall structure, we chose to examine gene-rifampicin interactions on solid media which has neither the shear forces or detergent nor the murine immune pressure as additional stresses. We also utilized *M. bovis* BCG Pasteur, which carries two duplicated genome regions (DU1 and DU2), serendipitously allowing investigation of rifampicin interactions with essential genes occurring in a merodiploid state in these regions.

Similar to previous studies we found a number of rifampicin sensitive insertions in genes involved in cell wall synthesis or in regulation of cell wall mechanisms, supporting the importance of cell wall integrity on rifampicin tolerance. Disruption of the Chp2 acyltransferase required for production of outer-membrane PATs rendered the bacterium sensitive to 1/250 the MIC of rifampicin. We also identified that mutations in IniB (Rv0341), a glycine-rich protein which is expressed with two bacterial dynamin-like proteins (IniA and IniC) and implicated in isoniazid and ethambutol resistance/sensitivity [41] conferred rifampicin sensitivity. It is likely that IniABC are involved in cell wall/plasma membrane stabilization [21] and that their absence increases permeability to multiple drugs. Indeed, we might consider this system an important stress response to antibiotics because previous work has also shown that the IniBAC operon is induced by various antibiotics including rifampicin [38,41] but this report is the first evidence that this membrane stabilization complex is involved in tolerance to rifampicin. Other systems that may alter the permeability of the bacterium included the putative ABC transporter Rv0986 [42] which is associated with changes in surface adhesion and virulence, but whose natural transport cargo has not been well characterized, but rather than directly transporting rifampicin may export cell wall components which alter permeability. It is of note that none of the expected putative drug efflux systems were indicated to be individually necessary for rifampicin resistance, although this may be due to redundancy.

Aside from mechanisms directly associated with permeability, this study identified several regulators of gene expression which may be involved in the regulation of cell wall structure and associated process (MceR1 controlling MCE1 transporter, RsmA controlling the ESX-4 secretory apparatus). Perhaps the most interesting of the regulatory molecules implicated in rifampicin tolerance was the PknE sensor kinase which may identify a pathway by which the bacterium can sense rifampicin or its effects and induce a tolerance-promoting stress response. Further work is needed to identify the signalling cascade from PknE stimulation.

As might be expected for an antibiotic which inhibits transcription of DNA to RNA, we identified genes involved in DNA damage repair as required for tolerance to rifampicin. Indeed, transcription itself is a source of DNA damage and here mutation of the scanning UvrA component of the UvrABC nucleotide excision repair complex renders the bacterium sensitive to low levels of rifampicin. This agrees with studies in *E. coli* which show hypersensitivity of UvrA and UvrB mutants to rifampicin [43,44]. We also identified Rnase HI activity as a requirement to resist rifampicin. The mechanisms that link Rnase HI deletion and rifampicin sensitivity have been defined and involve the persistence of R-loops during transcription [34]. Furthermore, the potential for drugs targeting Rnase HI to potentiate rifampicin action in mycobacteria has been recently shown [34] and RNase HI may also represent a way to specifically target antibiotic resistant strains of bacteria [45]. Our study provides further evidence to support this exciting drug development area.

A further aspect of DNA biology highlighted in our mutant screen was nucleoid segregation. Although not previously recognized in mycobacteria, rifampicin has been demonstrated to inhibit nucleoid segregation in various bacteria [46,48]. Here, because BCG is merodiploid for the essential partition protein ParB, we were able to examine the effects of mutation of one copy of *parB* on sensitivity to rifampicin. We detected a significant increase in sensitivity to rifampicin in bacteria with insertion in *parB* indicative of a close interaction between RNA polymerase and chromosome segregation.

In summary, the rifampicin-gene interaction screen described in the present study identifies genes involved in cell wall synthesis and maintenance, alongside regulatory pathways and nucleic acid metabolism genes which allow the bacteria to adapt to the stress of rifampicin treatment. Unlike previous screens of rifampicin sensitivity/tolerance/persistence, the present screen was not dominated by cell wall mutants, which may explain why other mechanisms more closely associated with the mechanism of action of rifampicin such as degradation of RNA loops, nucleotide excision repair or chromosome segregation were identified as important to innate resistance to the antibiotic. It is of note that BCG has significant genomic differences compared to *M. tuberculosis* including deletion of the ESX-1 secretion system, which has been linked to changes in cell wall lipid composition [49]. Thus, the difference between screens may result from cell wall differences between *M. tuberculosis* and *M. bovis* BCG (used here) or from the different selection protocols, here using solid media with very low sub-MIC levels of rifampicin. Future work will investigate further how these mechanisms may be targeted as adjunctive therapies to decrease the concentration or duration of rifampicin treatment.

## supplementary material

10.1099/mic.0.001488 Uncited Table S1.

## Abbreviations

BCG: Bacille Calmette-Guérin
MDR: multiple drug resistant
MIC: minimum inhibitory concentration
OADC: oleic acid dextrose ovalbumin
PAT: polyacyltrehalose
TB: Tuberculosis
